# A Big Catch for Germ Cell Tumour Research

**DOI:** 10.1371/journal.pgen.1003481

**Published:** 2013-04-11

**Authors:** Kenneth S. Chen, James F. Amatruda

**Affiliations:** 1 Department of Pediatrics, University of Texas Southwestern Medical Center, Dallas, Texas, United States of America; 2 Center for Cancer and Blood Disorders, Children's Medical Center, Dallas, Texas, United States of America; 3 Department of Molecular Biology, University of Texas Southwestern Medical Center, Dallas, Texas, United States of America; 4 Department of Internal Medicine, University of Texas Southwestern Medical Center, Dallas, Texas, United States of America; University of Utah, United States of America

**Figure 1:**
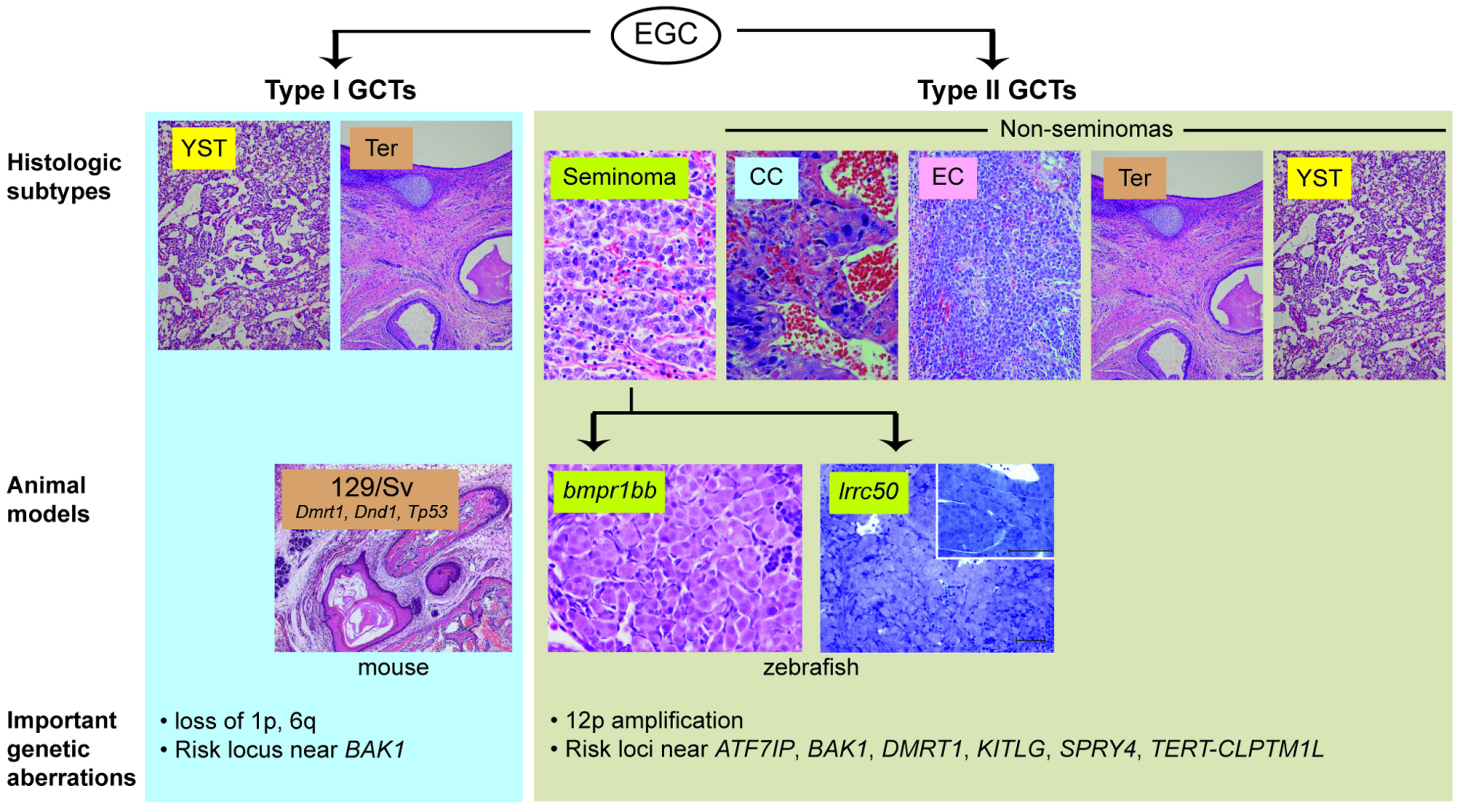
Animal models of TGCT and correlation to human histologic subtypes. TGCTs appear to arise from embryonic germ cells (EGC) in infants and young children (“type I”) or in adolescents and young adults (“type II”) [4]. (For clarity, the presumed carcinoma *in situ* precursor cell for type II tumours is omitted). These are further sub-divided by histologic subtype: yolk sac tumour (YST), teratoma (Ter), seminoma, choriocarcinoma (CC), embryonal carcinoma (EC). Current animal models of TGCT correlate to type I teratomas (129/Sv mice) and type II seminomas (*bmpr1bb* or *lrrc50* fish). Important genetic aberrations in human tumours are listed below. Mouse teratoma photo courtesy of Dr. David Zarkower.

Testicular germ cell tumour (TGCT) is the most common cancer in young men, and the incidence of TGCT is rising worldwide for unknown reasons [1], [2]. Treatments for TGCT are overall quite effective, but at the cost of significant toxicity [3], creating a powerful incentive for the development of more specific, molecularly guided therapies. TGCTs are generally thought to arise from a pluripotent fetal or embryonic germ cell [4]. Reflecting this pluripotency, these tumours can present in a wide range of histologic forms. Seminomas are TGCTs that retain features of pluripotent, primitive germ cells. In contrast, non-seminoma TGCTs exhibit differentiation into forms resembling somatic tissues (teratomas) or extraembryonic structures such as yolk sac (yolk sac tumour) or placenta (choriocarcinoma) (Figure 1). Family members of TGCT patients have a markedly increased risk of developing TGCT, strongly implicating an underlying genetic basis. Recent genome-wide association studies of TGCT have identified SNPs near ATF7IP, BAK1, DMRT1, KITLG, SPRY4, and TERT-CLPTM1L that increase TGCT risk [5]–[7]; however, the mechanisms associating most of these loci with tumourigenesis remain unclear.

Researchers seeking to identify such mechanisms have been hindered by the limited TGCT animal models available thus far. Two heritable TGCT models have been previously described: one in mouse and one in zebrafish. The mouse model arose through the observation by Leroy Stevens in the late 1950s that testicular teratomas arise spontaneously at low frequency during embryonic development in mice of the 129/Sv strain [8]. This discovery, which ultimately led to the experimental derivation of embryonic stem cells [9], [10], has also proved to be a useful model of teratoma formation. A number of genes have been identified as modifier loci that increase teratoma incidence in the 129/Sv background, including *Tp53*, *Dmrt1*, and *Dnd1*, an RNA-binding protein that is central to germ cell maintenance [11]–[13]. Forward genetic screening led to the discovery of a second *in vivo* TGCT model. Zebrafish carrying nonsense mutations in *alk6b/bmpr1bb*, an ortholog of the human bone morphogenetic protein (BMP) receptor *BMPR1B*, develop TGCTs resembling human seminomas [14], [15]. This finding illuminated the importance of BMP signaling in germ cell development and implicated disruption of BMP signaling in human germ cell tumourigenesis [16]. These two models have provided insight into the roles of pluripotency and differentiation pathways in TGCT development; however, their direct correlation to human tumourigenesis has been limited, as genes such as *DND1* and *BMPR1B* have not been found to be mutated in human TGCTs [14], [17].

In this issue of *PLOS Genetics*, Basten and coworkers describe a new zebrafish TGCT model with a direct connection to human TGCT mutations [18]. Zebrafish with homozygous mutations in the ciliary protein *lrrc50* were previously described to have kidney cysts homologous to human polycystic kidney disease [19]. In this paper, the authors report that male *lrrc50* heterozygotes develop testicular tumours late in life with near complete penetrance. Morphologically, these tumours, similar to those arising in *bmpr1bb-*deficient zebrafish, contain sheets of uniform, undifferentiated germ cells, resembling human seminoma. Loss of heterozygosity at the *lrrc50* locus was found in some tumours, consistent with a role of *lrrc50* as a tumour suppressor. The authors then conducted a mutational analysis of *LRRC50* in a collection of human seminoma samples and identified different mutations in two pedigrees with family history of seminomas, as well as heterozygosity for a different germline *LRRC50* mutation in five of 38 patients with sporadic seminomas. *LRRC50* is thus the first gene specifically linked to seminoma predisposition in humans. The mutations were found to be functional nulls through their inability to complement *lrrc50* knockdown in zebrafish embryos, an elegant example of the utility of the fish system for both gene discovery (forward genetics) and functional genomics (reverse genetics).

*lrrc50* has heretofore been characterized solely as a ciliary motor protein, and its connection to GCT suppression is intriguing. Cilia have not previously been thought to be present in spermatogonia, but the authors show that normal spermatogonia do indeed have a cilium and that LRRC50 colocalized with the axoneme in spermatogonial stem cells. In addition, the authors provide evidence that its role may not be solely structural by showing that its expression is cell cycle–regulated and that it localizes with condensed chromosomes. The development of both renal cysts in homozygotes and seminomas in heterozygotes may implicate an underlying role for *lrrc50* in early genitourinary development. Furthermore, the primary cilium has emerged as a signaling center for Hedgehog, Wnt, and other developmental pathways [20], and this, along with the TGCT phenotype of *bmpr1bb* mutants, raises the interesting possibility that interplay of these developmental signaling pathways is central to TGCT tumour suppression. Follow-up mechanistic studies will be critical to testing these hypotheses.

This paper provides another example of the power of zebrafish forward genetic screens for discovery of genes with novel roles in cancer and other diseases, and is a welcome addition to the list of animal models of TGCT. Some caveats apply when comparing analogous tumours arising in animals separated by a large evolutionary distance; for example, the fish seminomas are benign compared to human seminomas, and may well arise at a different stage of germ cell development. More significantly, neither the fish nor the mouse models currently reflect the biology of human non-seminomatous GCTs, such as embryonal carcinoma, choriocarcinoma, or yolk sac tumour. Clinically the non-seminomas are more likely to be metastatic and resistant to standard treatments, meaning that new models of these GCT subtypes are urgently needed to provide insight into tumour biology, as well as platforms for testing new therapeutic strategies for these cancers.
